# Supplementary material for: From sequence to enzyme mechanism using multi-label machine learning
Source: BMC Bioinformatics. 2014 May 19;15:150. doi: 10.1186/1471-2105-15-150 (PMC4229970; doi:10.1186/1471-2105-15-150)
Supplement: Additional file 2 — Java code of ml2db. Additional file ml2db_code.tar.gz contains the Java source code to run the multi-label machine learning experiments and save the results to database. The code’s Javadoc is included. [file 1471-2105-15-150-S2.zip › additional file 2/ml2db/ml2db/doc/mulan/evaluation/measure/class-use/RankingMeasureBase.html]

Uses of Class mulan.evaluation.measure.RankingMeasureBase


JavaScript is disabled on your browser.


- Overview
- Package
- Class
- Use
- Tree
- Deprecated
- Index
- Help

- Prev
- Next

- Frames
- No Frames

- All Classes

## Uses of Class mulan.evaluation.measure.RankingMeasureBase

- Packages that use RankingMeasureBase

  | Package | Description |
  | --- | --- |
  |  |  |
  | --- | --- |
  | mulan.evaluation.measure |  |
- - ### Uses of RankingMeasureBase in mulan.evaluation.measure

    Subclasses of RankingMeasureBase in mulan.evaluation.measure

    | Modifier and Type | Class and Description |
    | --- | --- |
    |  |  |
    | --- | --- |
    | `class` | `AveragePrecision` Implementation of the average precision measure. |
    | `class` | `Coverage` Implementation of the coverage measure. |
    | `class` | `ErrorSetSize` Measure based on the ErrorSetSize ranking loss function |
    | `class` | `IsError` Measure based on the IsError ranking loss function |
    | `class` | `LossBasedRankingMeasureBase` |
    | `class` | `OneError` Measure based on the one error loss function |
    | `class` | `RankingLoss` Implementation of the ranking loss measure. |

- Overview
- Package
- Class
- Use
- Tree
- Deprecated
- Index
- Help

- Prev
- Next

- Frames
- No Frames

- All Classes
